# Congenital portopulmonary shunt in a cat

**DOI:** 10.1111/jsap.13545

**Published:** 2022-09-04

**Authors:** K. Terai, K. Ishigaki, Y. Sakamoto, N. Sakurai, T. Heishima, O. Yoshida, M. Sakai, K. Asano

**Affiliations:** ^1^ Laboratory of Veterinary Surgery, Department of Veterinary Medicine, College of Bioresource Sciences Nihon University Fujisawa Kanagawa Japan; ^2^ Laboratory of Veterinary Hepatology & Gastroenterology, Department of Veterinary Medicine, College of Bioresource Sciences Nihon University Fujisawa Kanagawa Japan

## Abstract

A 9‐year‐old spayed female crossbreed cat with chief complaints of anorexia and hypersalivation had high serum concentrations of ammonia and fasting and postprandial total bile acid. Therefore, she was referred to our hospital. On the first evaluation, haematology, serum chemistry, radiography and ultrasonography findings suggested that she had a congenital portosystemic shunt. CT revealed a shunt vessel from the left gastric vein to the left pulmonary vein. During median celiotomy and sternotomy, gross findings and mesenteric portography revealed abnormal vessel shunting from the left gastric vein to the left pulmonary vein. Complete ligation of the shunt vessel was achieved. She recovered without any complications. Postoperative serum chemistry revealed that ammonia and total bile acid levels decreased to within the reference intervals. This report is the first to describe the clinical features and surgical outcome of a cat with a congenital portopulmonary shunt.

## INTRODUCTION

A portosystemic shunt (PSS) is an anomalous vessel that allows portal blood draining from the stomach, intestines, pancreas and spleen to pass directly into the systemic circulation without first passing through the liver (Tivers & Lipscomb 2011). Congenital PSSs in cats have an incidence of 2.5 per 10,000 cats treated in referral centres (Levy *et al*. 1995). Congenital PSS can be extrahepatic or intrahepatic, with the extrahepatic type be more common (Tivers & Lipscomb 2011).

Previous studies (White *et al*. 2017, Radlinsky & Fossum 2018, Valiente *et al*. 2019) have described morphological variations of feline congenital extrahepatic PSS in which the shunt inserts on the caudal vena cava, phrenic vein or azygous vein. However, no report has described a congenital shunt between the portal venous system and the pulmonary circulation, which should be called a congenital portopulmonary shunt (PPS) and differs from congenital PSS. This report describes the clinical features and surgical outcome of a cat with congenital PPS.

## CASE HISTORY

**FIG 1:**
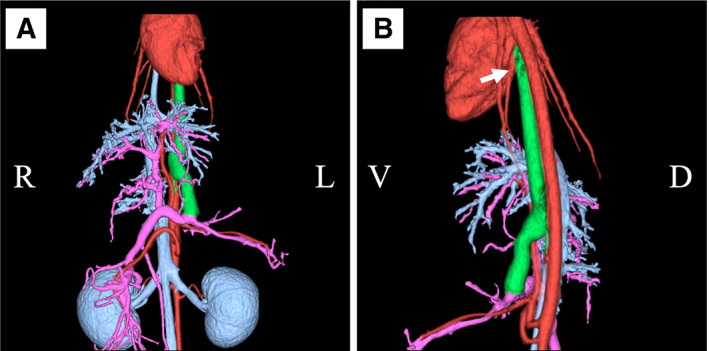
CT angiography findings. Green indicates the abnormal shunt blood vessel; purple, the portal vein system; and light blue, the venous system and kidney. Red indicates the heart, aorta, pulmonary artery and pulmonary vein. (A) The ventral side. The shunt vessel (green) continues from the left gastric vein to the dorsal side of the heart. (B) The left side. The shunt vessel (green) joins the left pulmonary vein (arrow)

A 9‐year‐old spayed female crossbreed cat, weighing 3.2 kg, was referred to our hospital with suspected congenital PSS due to anorexia and hypersalivation, indicative of hepatic encephalopathy. The cat had hyperammonaemia (598 μg/dL; reference interval: 23 to 78 μg/dL) and high levels of serum total bile acid (TBA) (fasting: 11.2 μmol/L; reference interval: 0.0 to 5.0 μmol/L; postprandial: 79.3 μmol/L; reference interval: 0.0 to 15.0 μmol/L) at the time of examination at the referral hospital. On the first evaluation, the cat had a normal body condition score. The cat had pale mucous membranes on physical examination but no findings indicative of hepatic encephalopathy. No cardiac murmurs were auscultated. The serum chemistry results revealed increased levels of liver enzymes, ammonia, TBA and serum amyloid A, and decreased levels of albumin and total cholesterol (Table 1). Blood coagulation tests indicated decreased antithrombin activity and prolonged activated partial thromboplastin time (APTT) (Table 1). Thoracic radiography revealed no obvious enlargement of the cardiac silhouette (vertebral heart size: 7.6). Abdominal radiography revealed microhepatia. Abdominal ultrasound revealed a reduced portal vein diameter (1.1 mm) at the hepatic hilum. The diameter of the caudal vena cava was 4.7 mm at the same level. Therefore, the ratio of the portal venous diameter to the caudal vena cava diameter was 0.23, using the calculation method described in a previous study (D'Anjou *et al*. 2004). Furthermore, no abnormal intrahepatic veins were visible; therefore, abdominal ultrasound findings suggested congenital extrahepatic PSS. CT angiography (CTA) findings revealed that the cat had a single extrahepatic shunt vessel connecting the left gastric vein to the left pulmonary vein (Fig 1). The shunt vessel originated from the left gastric vein, ran along the dorsal side of the gastric cardia, entered into the thoracic cavity, and terminated in the left pulmonary vein originating from the left caudal lung lobe (i.e. immediately before the inflow of the left atrium). The intrahepatic portal venous flow was observed and was confirmed up to its fourth branches. The cat was managed medically with lactulose (Kowa Co., Ltd., Nagoya, Japan) and metronidazole (Flagyl; Shionogi and Co., Ltd., Osaka, Japan) until surgery 36 days later.

**Table 1:** R blood test

Parameter	Unit	Perioperati	Postoperati	Reference interval
RBC	10^6^/μL	7.3	5.9	5.0 to 10.0
PCV	%	32	26	24 to 45
WBC	/μL	11,800	11,500	5500 to 19,500
Plt	10^3^/μL	197	283	300 to 800
TP	g/dL	6.5	6	5.7 to 7.8
Alb	g/dL	1.9	2.3	2.3 to 3.5
Glu	mg/dL	104	116	71 to 148
TCho	mg/dL	87	127	95 to 259
AST	U/L	52	24	18 to 51
ALT	U/L	71	29	22 to 84
ALP	U/L	75	43	0 to 58
GGT	U/L	0	1	0 to 10
TBil	mg/dL	0.3	0	0.0 to 0.4
NH_3_	μg/dL	99	67	23 to 78
TBA (fasting)	μmol/L	8.0	NA	0.0 to 5.0
BUN	mg/dL	18.8	22.1	17.6 to 32.8
Cr	mg/dL	1.33	1.3	0.9 to 2.1
Na	mEq/L	150	156	147 to 156
K	mEq/L	3.4	3.6	3.4 to 4.6
Cl	mEq/L	118	119	107 to 120
SAA	μg/mL	11.2	5.4	0.0 to 5.5
APTT	second	54.8	NA	10 to 32
PT	second	7.5	NA	6 to 8
Fib	mg/dL	179.2	NA	52 to 302
AT	%	90	NA	107 to 141
D‐dimer	μg/mL	0.38	NA	0.0 to 1.5

Postoperative day 13, RBC Red blood cell count, PCV Packed cell volume, WBC White blood cell count, Plt Platelet count, TP Total protein, Alb Albumin, Glu Glucose, TCho Total cholesterol, AST Aspartate aminotransferase, ALT Alanine aminotransferase, ALP Alkaline phosphatase, GGT Gamma‐glutamyltransferase, TBil Total bilirubin, NH_3_ Ammonia, TBA Total bile acid, BUN Blood urea nitrogen, Cr Creatinine, SAA Serum amyloid A, APTT Activated partial thromboplastin time, PT Prothrombin time, Fib Fibrinogen, AT Anti‐thrombin activity, NA Not analysed.

Subcutaneous (sc) injections of 0.04 mg/kg atropine (Mitsubishi Tanabe Pharma Co., Osaka, Japan), 1.0 mg/kg prednisolone (Kyoritsu Seiyaku Co., Tokyo, Japan), 1.0 mg/kg famotidine (Gaster; LTL Pharma Co., Ltd., Tokyo, Japan), and 1.0 mg/kg maropitant citrate (Cerenia; Zoetis, Parsippany, NJ), and an intravenous (iv) injection of 20 mg/kg levetiracetam (E Keppra; UCB Japan Co., Ltd., Tokyo, Japan) were used as premedications. Ampicillin [20 mg/kg (iv); Viccillin; Meiji Seika Pharma Co., Ltd., Tokyo, Japan] was administered as the perioperative antibiotic. General anaesthesia was then induced with 3 mg/kg propofol (Mylan; Mylan Seiyaku Ltd.).

After endotracheal intubation, the cat was mechanically ventilated with a mixture of isoflurane (IsoFlo; Zoetis) and oxygen. For intraoperative medical management, dopamine (2.5 to 5 μg/kg/minute; Teva Takeda Pharma Ltd., Nagoya, Japan) and dobutamine (0.5 to 5 μg/kg/minute; Kyowa Pharmaceutical Industry Co., Ltd., Osaka, Japan) were continuously infused for hypotension. For analgesia, the cat was administered an intraoperative and postoperative continuous butorphanol (Betorphal; Meiji Seika Pharma Co., Ltd.) continuous rate infusion.

**FIG 2:**
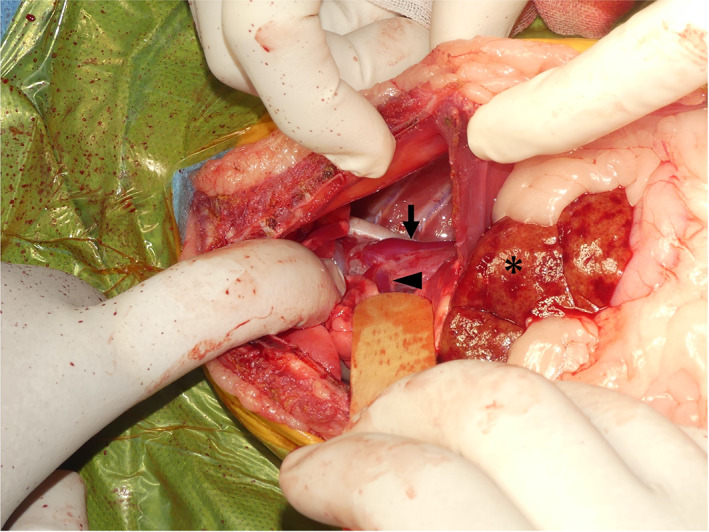
Intraoperative photograph. The thoracic cavity after the median sternotomy and diaphragmatic incision. The shunt blood vessel (arrow) in the thoracic cavity can be seen flowing into the left pulmonary vein (arrowhead), originating from the left caudal lung lobe. The liver (asterisk) is small

The cat was positioned in dorsal recumbency. A celiotomy was performed from the xiphoid to the middle between the umbilicus and pubis. The gross findings revealed a small liver with suspected hypoplasia. Its surface was slightly yellowish with irregular dark red spots. The superficial omental wall was bluntly dissected, and the left gastric vein was confirmed. In addition, a caudal median sternotomy was performed, followed by a diaphragmatic midline incision. An abnormal vessel running in the caudal mediastinum was observed (Fig 2).

**FIG 3:**
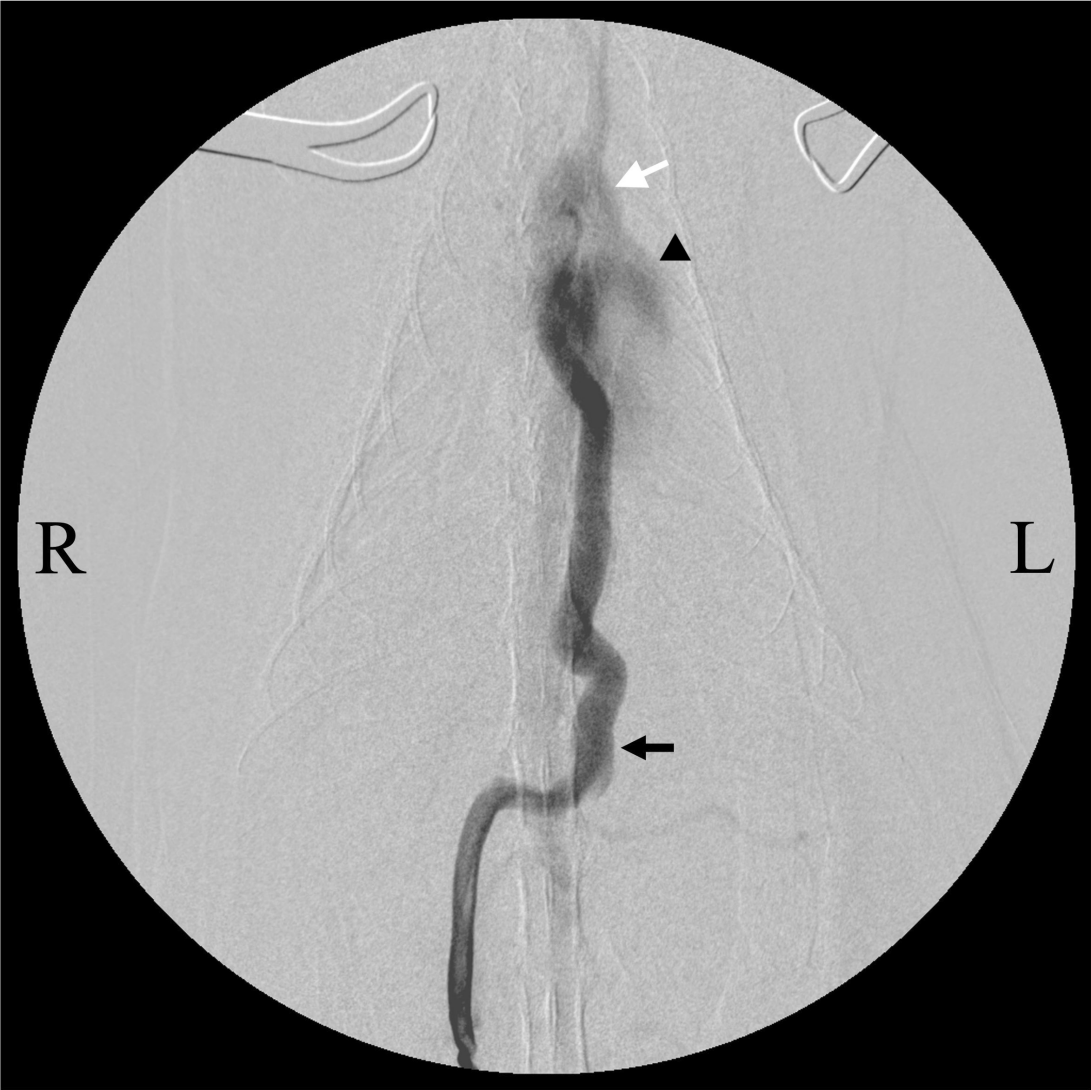
Findings of intraoperative mesenteric portography. The contrast medium, injected from the jejunal vein, flows from the left gastric vein (black arrow) to the left pulmonary vein, and enters the left atrium (black arrowhead). It flows to the aorta (white arrow) via the left ventricle

A jejunal vein was cannulated to monitor portal pressure and perform mesenteric portography (MPG). The measured portal pressure was 7 mmHg. A subsequent MPG demonstrated that portal blood flowed through the left gastric vein via the shunt vessel and into the left pulmonary vein originating from the left caudal lung lobe with little portal inflow to the liver (Fig 3).

**FIG 4:**
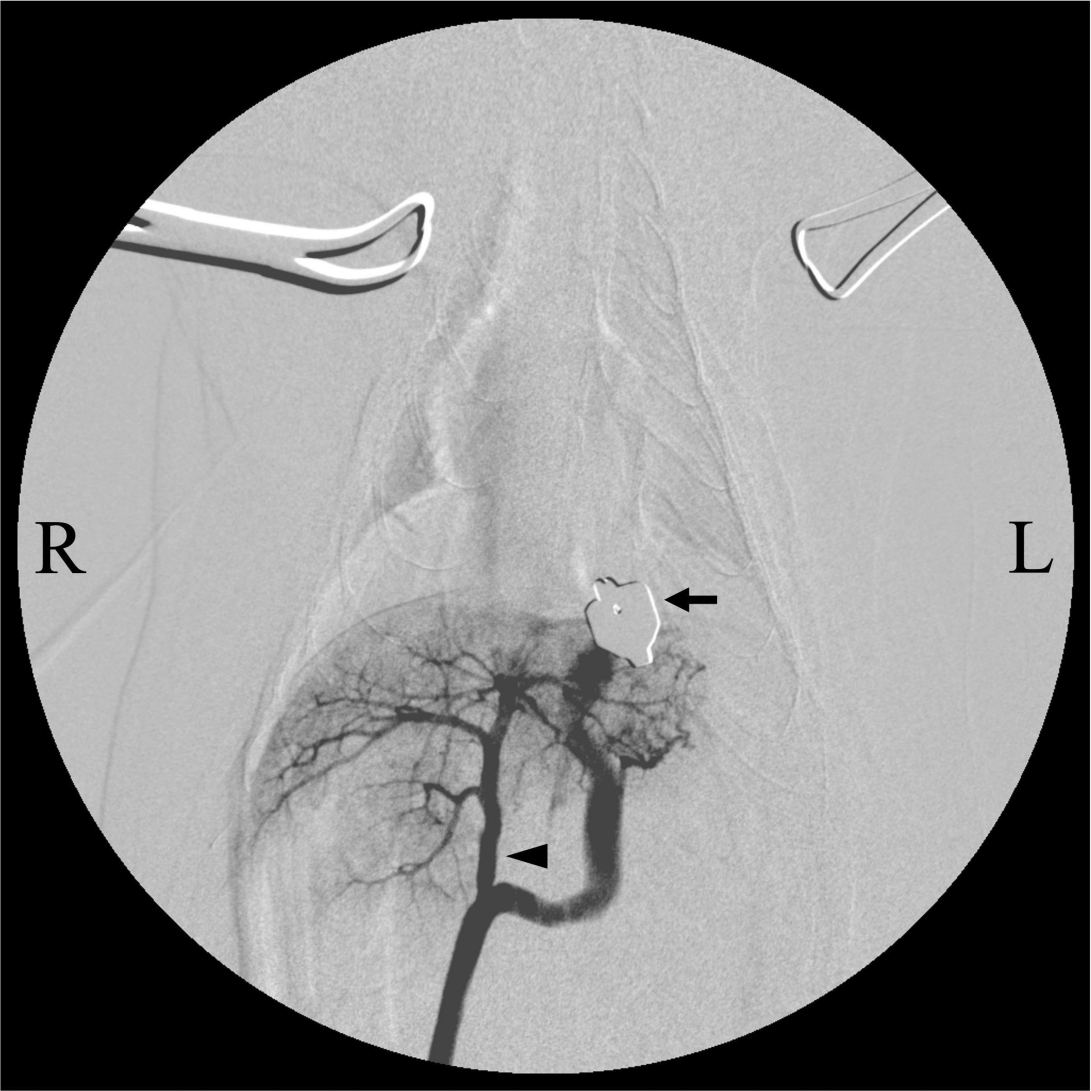
Findings of intraoperative mesenteric portography after temporary occlusion. Temporary occlusion is achieved in the thoracic cavity with atraumatic bulldog forceps (arrows). Contrast medium, injected from the jejunal vein, flows into the liver through the original portal vein (arrowhead). No other shunt vessels are visible

The shunt vessel was macroscopically identified as connecting to the left pulmonary vein originating from the left caudal lung lobe. The shunt vessel running in the left ventral side of the oesophagus was isolated approximately halfway between the heart and diaphragm by using right‐angled forceps. During temporary occlusion of the isolated shunt vessel, MPG demonstrated sufficient intrahepatic portal blood flow up to the fourth branches without any flow in the shunt vessel (Fig 4). During the temporary occlusion, the portal pressure increased to 9 mmHg, and no changes occurred in the colour of the gastrointestinal tract and pancreas, intestinal peristalsis, and vital signs, including heart rate and end‐tidal carbon dioxide. After the temporary occlusion was released, the shunt vessel was completely ligated at the same site as the temporary occlusion with a coated braided nylon suture, size 0 (Surgilon; Medtronic Inc., Minneapolis, MN). A wedge excisional liver biopsy using the vessel sealing system was performed for histopathologic examination.

A chest tube was placed through the abdominal wall and diaphragm into the thoracic cavity. The incised diaphragm was sutured using a continuous pattern with 2‐0 absorbable sutures (PDS II; Johnson and Johnson, New Brunswick, NJ). The median sternotomy was closed using an interrupted cruciate pattern with absorbable sutures, size 0 (PDS II). The abdominal wall, subcutaneous tissues, and skin were routinely closed.

On postoperative day 1 (POD 1), levetiracetam [20 mg/kg (iv); E Keppra; UCB Japan Co., Ltd.] was administered three times daily to prevent postoperative seizures, and ampicillin [20 mg/kg (sc); Viccillin] was administered twice daily as an antibiotic. No lactulose was administered postoperatively because of normalised blood ammonia level (68 μg/dL) on POD 1. A postoperative continuous drip infusion of 0.06 mg/kg/hour butorphanol was maintained until 3 days after surgery. On POD 2, the cat's appetite recovered and 19.5 mg/kg twice a day oral levetiracetam (E Keppra; UCB Japan Co., Ltd.) and 19.5 mg/kg twice a day oral amoxicillin/clavulanic acid (Augmentin; Glaxo Smith Kline plc., Brentford, UK) were changed from the intravenous medications. The chest tube was removed on POD 3, and no postoperative neurological signs or suspected portal hypertension were found during the hospitalisation. The cat was discharged on POD 6 and administration of levetiracetam and amoxicillin/clavulanic acid was discontinued. At that time, the blood ammonia level was 29 μg/dL.

Hepatic histopathology findings included mild irregularity of the lobular structure, moderate to marked decrease in the inner diameter of the portal vein, hyperplasia of arterioles and bile ducts and mild vacuolar degeneration of hepatocytes, which are typical histopathologic findings in cats with congenital PSS (Tivers & Lipscomb 2011). On POD 13, the routine physical examination findings were within normal limits, and serum chemistry findings revealed no abnormalities (Table 1). Suture removal was performed.

On POD 107, the cat's general condition was good, and the serum chemistry findings revealed no abnormalities in the levels of ammonia (54 μg/dL) and TBA (fasting: 2.2 μmol/L; postprandial: 3.2 μmol/L). Thoracic and abdominal radiography revealed no postoperative complications, although microhepatia remained.

## DISCUSSION

Various forms of congenital extrahepatic PSS in cats have been reported (White *et al*. 2017, Radlinsky & Fossum 2018, Valiente *et al*. 2019). However, this paper is the first case report of a congenital PPS (i.e. left gastric‐left pulmonary vein shunt) in a cat. The first human case of congenital portal vein inflow into the pulmonary vein as a variant of the Abernethy malformation was treated with the closure of the shunt vessel (Sieverding *et al*. 2022). This human patient had symptomatic hyperammonaemia and arterial desaturation because of persistent pulmonary hypertension that was resistant to medical management. Our feline patient had no findings suggestive of pulmonary hypertension. However, pulmonary hypertension may require attention in cases of congenital PPS because of the increased volume in the pulmonary circulation.

The portal vein embryologically develops from the right yolk sac vein. The posterior vena cava develops from the right yolk sac vein, the caudal cardinal vein, the subcardinal vein, and the supracardinal vein, depending on the location. In carnivores, the azygos vein develops from the right supracardinal vein (Hyttel 2010). Congenital extrahepatic PSS occurs because of abnormal anastomosis between the vitelline venous system and the cardinal venous system (Payne *et al*. 1990). Pulmonary veins develop from the pulmonary venous plexus, which develops around the lung buds. The pulmonary venous plexus is reticulated with the circulatory system and the splanchnic venous plexus. Blood from the lungs flows through the splanchnic venous plexus into the cardinal vein, umbilical vein, and vitelline vein. As development progresses, the pulmonary venous plexus connects to the common pulmonary veins that have erupted from the left atrium. Blood flow between the splanchnic venous plexus is then cut off so that blood from the lungs flows only to the left atrium (Edwards 1953, Neill 1956). Hence, the abnormality in the current case may have occurred because of a residual or abnormal anastomosis of the pulmonary venous plexus and splanchnic venous plexus during the embryonic period. A previous report (Inglés *et al*. 1993) also suggests that cats with portal hypertension have secondary collaterals to the pulmonary veins. Nonfunctional communication may occur between the pulmonary vein and the portal vein. This nonfunctional communication may then open and remain during embryological development.

A shunt vessel from the portal venous system to the pulmonary circulation, as in our patient, has not been described in veterinary literature; therefore, the details of the shunt morphology are unknown. In our patient, the shunt vessel was visible through the epiploic foramen during celiotomy. Therefore, surgical ligation may be possible without a caudal median sternotomy. However, the possibility of other small shunt vessels that could not be confirmed with contrast‐enhanced CT or MPG was considered. Therefore, we added a caudal median sternotomy, and identified the complete morphology of the shunt vessel from the left gastric vein to the left pulmonary vein. In the future, when patients with this type of shunt morphology are treated, only celiotomy may be sufficient for the surgical ligation of the shunt vessel as the surgical approach.

Anaesthesia was carefully planned because this shunt morphology had not been reported previously. Butorphanol was used for analgesia, as cats experience an opioid‐induced increase in sympathetic outflow and central stimulatory effects (Bortolami & Love 2015). The cat recovered its appetite on POD 2 and appeared calm and relaxed. We have now started to use mu‐opioids as analgesics for this type of surgery and will use them cautiously in similar cases in the future. In addition, postoperative neurological complications are the most important complications to recognise; we opted to add preoperative prednisolone to the cat's anaesthetic protocol as it is generally used for preventing hypoglycaemia and neurological problems in the perioperative period in our hospital. In addition, levetiracetam was administered for postoperative seizure prophylaxis and was started preoperatively to increase postoperative blood levels.

Most cats with congenital extrahepatic PSS develop signs associated with hepatic encephalopathy within the first 6 months of life. However, some cats may not show clinical signs until maturity, or shunts may be diagnosed incidentally (Tivers & Lipscomb 2011). In our patient, hypersalivation suggestive of hepatic encephalopathy was observed at 9 years old. Systemic venous pressure in cats is between 1.5 and 3.7 mmHg (Hall 2016). Pulmonary artery incision wedge pressure, which indirectly represents the pulmonary venous pressure, ranges 3 to 12 mmHg in various animals (Dula *et al*. 1985, Dermlim *et al*. 2019, Wise *et al*. 2021, Nair & Lamaa 2022), and pulmonary venous pressure is generally higher than systemic venous pressure. Therefore, the difference from the portal vein pressure is smaller in pulmonary venous pressure than in systemic venous pressure, and it may cause a lower shunt fraction in congenital PPS than in congenital PSS.

Previous reports have suggested that portophrenic and portoazygous shunts are compressed by increased intrathoracic pressure during the respiratory cycle, thereby decreasing the shunt fraction and consequently resulting in fewer clinical signs in dogs (Kraun *et al*. 2014, Amaha *et al*. 2019). In our feline patient, the shunt fraction may have decreased during expiration by the same mechanism. Therefore, our case suggested that congenital PPS may be clinically detected at an older age, compared to congenital PSS, because of the low shunt fraction.

The clinical signs and blood test findings in this patient were similar to those of congenital extrahepatic PSS (Tivers & Lipscomb 2011, Tzounos *et al*. 2017). Hypersalivation occurs in 67% to 84% of congenital extrahepatic PSS patients (Tivers & Lipscomb 2011). Elevated liver enzymes and decreased liver function are common in congenital extrahepatic PSS (Tivers & Lipscomb 2011). Our patient had a prolonged APTT, but no intraoperative bleeding tendency. A previous report (Tzounos *et al*. 2017) similarly demonstrated that cats with congenital extrahepatic PSS have a prolonged APTT but no bleeding tendency. Therefore, the pathological condition of congenital PPS is similar to that of congenital extrahepatic PSS with similar clinical signs and serum chemical profiles.

In this patient, preoperative radiography and ultrasonography findings revealed microhepatia, which is less common in cats (Radlinsky & Fossum 2018), and shrinking of the portal vein in the hepatic hilum (D'Anjou *et al*. 2004). Congenital extrahepatic PSS was suspected but was not definitively diagnosed. The extrahepatic portal vasculature and anatomy of the shunt vessels in congenital extrahepatic PSS can be visualised with CTA (Parry & White 2017). A diagnosis of a congenital PPS can be made based on CTA, while the images are also useful for preoperative surgical planning. In this patient, a shunt vessel between the left gastric and the pulmonary veins was diagnosed based on CTA findings.

In conclusion, here we report the first case of a congenital PPS (i.e. left gastric to left pulmonary vein shunt) in a cat. Clinical features and characteristics of laboratory and ultrasonographic examinations of congenital PPS may be similar to those of congenital extrahepatic PSS. However, CTA is useful for their differential diagnosis. The prognosis for congenital PPS may be good with the same treatment used for congenital extrahepatic PSS.

### Author contributions

**K. Terai:** Conceptualization (supporting); data curation (supporting); investigation (supporting); methodology (supporting); visualization (lead); writing – original draft (equal); writing – review and editing (equal). **K. Ishigaki:** Conceptualization (supporting); data curation (supporting); investigation (supporting); methodology (supporting); project administration (supporting); supervision (supporting); visualization (supporting); writing – original draft (equal); writing – review and editing (equal). **Y. Sakamoto:** Conceptualization (supporting); data curation (supporting); investigation (supporting); writing – original draft (equal); writing – review and editing (equal). **N. Sakurai:** Data curation (supporting); investigation (supporting); methodology (supporting); visualization (supporting); writing – original draft (equal); writing – review and editing (equal). **T. Heishima:** Data curation (supporting); investigation (supporting); methodology (supporting); visualization (supporting); writing – original draft (equal); writing – review and editing (equal). **O. Yoshida:** Conceptualization (supporting); data curation (supporting); investigation (supporting); methodology (supporting); project administration (supporting); supervision (supporting); writing – original draft (equal); writing – review and editing (equal). **M. Sakai:** Conceptualization (supporting); data curation (supporting); investigation (supporting); writing – original draft (equal); writing – review and editing (equal). **K. Asano:** Conceptualization (lead); data curation (lead); investigation (lead); methodology (lead); project administration (lead); supervision (lead); visualization (supporting); writing – original draft (equal); writing – review and editing (equal).

### Conflict of Interest

None of the authors of this article has a financial or personal relationship with other people or organisations that could inappropriately influence or bias the content of the paper.
